# Ethical issues in pragmatic randomized controlled trials: a review of the recent literature identifies gaps in ethical argumentation

**DOI:** 10.1186/s12910-018-0253-x

**Published:** 2018-02-27

**Authors:** Cory E. Goldstein, Charles Weijer, Jamie C. Brehaut, Dean A. Fergusson, Jeremy M. Grimshaw, Austin R. Horn, Monica Taljaard

**Affiliations:** 1 Rotman Institute of Philosophy, Western University, 1151 Richmond St., London, ON N6A 5B7 Canada; 2 Clinical Epidemiology Program, Ottawa Hospital Research Institute, Ottawa, ON Canada

**Keywords:** Pragmatic randomized controlled trials, Literature review, Ethics, Informed consent, Disclosure, Oversight, Risk, Regulation, Research ethics committees

## Abstract

**Background:**

Pragmatic randomized controlled trials (RCTs) are designed to evaluate the effectiveness of interventions in real-world clinical conditions. However, these studies raise ethical issues for researchers and regulators. Our objective is to identify a list of key ethical issues in pragmatic RCTs and highlight gaps in the ethics literature.

**Methods:**

We conducted a scoping review of articles addressing ethical aspects of pragmatic RCTs. After applying the search strategy and eligibility criteria, 36 articles were included and reviewed using content analysis.

**Results:**

Our review identified four major themes: 1) the research-practice distinction; 2) the need for consent; 3) elements that must be disclosed in the consent process; and 4) appropriate oversight by research ethics committees. 1) Most authors reject the need for a research-practice distinction in pragmatic RCTs. They argue that the distinction rests on the presumptions that research participation offers patients less benefit and greater risk than clinical practice, but neither is true in the case of pragmatic RCTs. 2) Most authors further conclude that pragmatic RCTs may proceed without informed consent or with simplified consent procedures when risks are low and consent is infeasible. 3) Authors who endorse the need for consent assert that information need only be disclosed when research participation poses incremental risks compared to clinical practice. Authors disagree as to whether randomization must be disclosed. 4) Finally, all authors view regulatory oversight as burdensome and a practical impediment to the conduct of pragmatic RCTs, and argue that oversight procedures ought to be streamlined when risks to participants are low.

**Conclusion:**

The current ethical discussion is framed by the assumption that the function of research oversight is to protect participants from risk. As pragmatic RCTs commonly involve usual care interventions, the risks may be minimal. This leads many to reject the research-practice distinction and question the need for informed consent. But the function of oversight should be understood broadly as protecting the liberty and welfare interest of participants and promoting public trust in research. This understanding, we suggest, will focus discussion on questions about appropriate ethical review for pragmatic RCTs.

## Background

Randomized controlled trials (RCTs) are a key method in the rigorous evaluation of health service delivery and medical treatments. RCTs are heterogeneous in both purpose and design, and they span the continuum from explanatory to pragmatic. As Schwartz and Lellouch describe, the objective of an explanatory RCT is *understanding*: to discover the efficacy of an intervention in ideal circumstances [1]. In contrast, the objective of a pragmatic RCT is *decision-making*: to evaluate the effectiveness of an intervention in usual clinical conditions [1]. In practice, the vast majority of trials cannot be categorized as wholly explanatory or pragmatic. Thorpe and colleagues argue that RCTs commonly have aspects of their design that are explanatory, while other aspects are pragmatic [2]. This is reflected in the PRagmatic Explanatory Continuum Indicator Summary 2 (PRECIS-2) tool created to aid trialists in the design of RCTs [3].

There is a growing international call for more pragmatic RCTs to evaluate health service delivery and treatments to inform decision-making by patients, clinicians, and managers [4, 5]. Several factors contribute to the increasing use of pragmatic RCTs. First, additional public research funds have been made available to support pragmatic RCTs. While explanatory trials are commonly sponsored by pharmaceutical companies, pragmatic RCTs are typically funded by government or public agencies [5]; in fact, facing rising health care costs, governments and public agencies have invested billions of dollars in patient-oriented and comparative effectiveness research (e.g., Canada’s Strategy for Patient-Oriented Research, the United States’ American Recovery and Reinvestment Act, and the United Kingdom’s Health Technology Assessment Programme) [6–8]. Second, the availability of electronic data systems and infrastructure that streamlines data collection facilitate the conduct of pragmatic RCTs in hospitals and clinics [9]. Third, and finally, the development of innovative pragmatic RCTs designs (e.g., cluster crossover trials and stepped-wedge cluster trials) opens the door for addressing questions in a novel way [10, 11].

Ethical issues and research regulations are widely perceived as obstacles to the design, review and conduct of pragmatic RCTs [4, 12]. Existing ethical and regulatory frameworks were developed with explanatory RCTs in mind, in which novel drugs are tested in tightly controlled conditions with individual patient recruitment, randomization, and follow-up. Traditional ethical principles underlying research are respect for persons, beneficence, justice and respect for communities [13, 14]. These ethical principles inform research regulations that require the up-front review of RCTs by research ethics committee. Research ethics committees (RECs) ensure that participant selection procedures are fair, study participation poses an acceptable balance of benefits and harms, and participants provide their informed consent.

Pragmatic RCTs differ in important ways from the explanatory RCTs that served as the model for these frameworks. In pragmatic RCTs, study interventions are often not experimental but treatments used routinely in practice; health providers in ordinary clinical settings provide care; and, data are collected from the electronic health record. These differences have prompted some to question the suitability of the traditional ethical framework for such studies, and whether a novel framework is required [15]. Others question whether the protections of research ethics committee review and informed consent are required for all pragmatic RCTs [16]. Finally, the application of research regulations to pragmatic RCTs may not be straightforward. For instance, when a trial involves usual care interventions should they be considered part of the research [17]? Usual care interventions are “treatments of procedures that have been accepted by medical experts as appropriate treatments or procedures for a given type of disease or condition, and are commonly used by healthcare professionals” [18].

If socially important research is to proceed, researchers, RECs and funders need guidance on ethical issues raised by pragmatic RCTs. The first step towards such guidance is the development of a complete picture of the ethical issues raised by pragmatic RCTs. Thus, the objectives of this scoping review are 1) to identify a list of key ethical challenges related to pragmatic RCTs from the ethics and trials literature, and 2) to highlight any gaps in the issues identified to date.

## Methods

We conducted a scoping review to identify articles relevant to the ethics of pragmatic RCTs [19]. A search was performed in PubMed on January 18, 2017 utilizing the terms “ethic,” “pragmatic” “comparative effectiveness,” “randomized controlled trial” “research,” and “clinical trial.” Articles published prior to January 2012 were excluded to maintain the focus on the literature arising from recent funding initiatives for pragmatic RCTs [6, 7]. Articles were also excluded if they were not in English or did not include discussion of ethical issues. We supplemented the electronic search by manually screening the references in a recent systematic review [20].

All selected articles were reviewed using content analysis. A descriptive analytical method was utilized to develop a summary description of the main themes. Each article was reviewed and given both a description and a related central theme. A determination was made by one author (CEG), and reviewed by a second author (CW), about whether the theme fit into an existing category or if a new category was required. This process resulted in a list of themes, which were presented and further distilled at three meetings with all authors. We discussed the alignment of the themes with the traditional research ethics framework, and we noted any discordance or gaps.

## Results

**Fig. 1:**
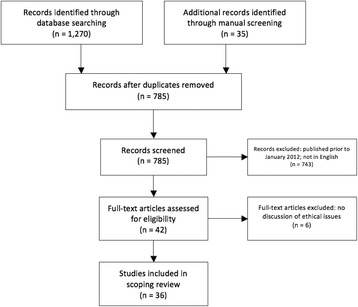
Electronic search strategy and article screening

Figure 1 summarizes the results from our search strategy. After applying the eligibility criteria, 36 articles were included in the review. Of the 36 articles, 35 were published in U.S. journals and 1 article was published in a UK journal.

### Overview of main themes

Table 1 summarizes four key themes related to ethical challenges in pragmatic RCTs: the research-practice distinction, the need for consent, elements that must be disclosed in the consent process, and appropriate oversight by RECs or other mechanisms. As discussed above, contemporary ethical and regulatory frameworks were developed with explanatory RCTs in mind, and pragmatic RCTs differ in important ways from them. These differences have prompted some authors to criticize the conventional research-practice distinction and conclude that the distinction does not apply to pragmatic RCTs [15, 16, 21–24]. They argue that the need for a research-practice distinction rests on the presumptions that research participation offers patients less benefit and greater risk than clinical practice, but that neither is true in the case of pragmatic RCTs. This leads to questions about whether such activities should be regulated as research or practice [21].

**Table 1:** Sumary of major themes

Main Themes	Associated Question	Majority View
Research-Practice Distinction	Is research morally distinct from clinical practice?	• Rejects the distinction between research and practice • Rests on an empirical claim that research often introduces risks that are unrelated to a patient’s clinical care • Problematic assumption that research automatically involves higher risks than clinical practice.
Consent	Is consent required, and if so how extensive must the consent process be for low-risk pragmatic RCTs?	• Low-risk pragmatic RCTs may proceed without consent (i.e., waiver of consent) • Limited to cases in which risk of participation is low and other consent options are unworkable • Altered informed consent models may strike a balance between autonomy and burden for researchers.
Disclosure	What aspects of research must be disclosed to research participants of low-risk pragmatic RCTs?	• Information should only be disclosed if research participation adds risks over and above clinical practice • Disagreement whether to disclose randomization • No disclosure required in an ethically robust learning health care system.
Oversight	What level of oversight is required for low-risk pragmatic RCTs?	• Oversight is time consuming, costly and complex • Burdens stem from the faulty research-practice distinction and the lack of inter-institutional standardization • Review process for low-risk pragmatic RCTs should be streamlined.

Authors debate the need for consent in pragmatic RCTs. The majority question whether consent is needed in pragmatic RCTs. According to international guidelines, a research ethics committee may grant a waiver of consent when risks to study participants are minimal, when the research has important social value, and requiring consent would render the study infeasible [25]. These authors assert that conditions for a waiver of consent commonly apply to pragmatic RCTs [16, 24, 26]. Other authors argue that consent is generally required in these studies [27–33]. Of those who believe consent is required, some claim that consent can be obtained in ways that better fit the clinical settings in which pragmatic trials are carried out [27–29].

Among authors who emphasize the need for informed consent, most agree that detailed information about the trial need only be disclosed when research participation involves incremental risks compared to clinical practice [27, 28, 34–38]. Authors agree that the purpose of the study, risks and benefits, and voluntary participation should be disclosed, but disagree as to whether randomization must be disclosed [27, 28, 34–39].

Finally, there is consensus that oversight by RECs is time consuming, costly, and overly complex, and, as a result, oversight is an impediment to research. Many authors believe that existing oversight procedures should be streamlined [40–44], while a few argue for a wholly new system of oversight for pragmatic RCTs [15].

### Elaboration of main themes

#### Research-practice distinction

There is consensus that one of the purposes of pragmatic RCTs is to “bridge the gaps in evidence” to provide patients with the best care [4, 20, 40, 45, 46]. Sugarman and Califf observe that “some patients do not receive the best care possible, either because research to support clinical decision making with high-quality evidence is lacking or because evidenced-based practices are not routinely implemented” [46]. The authors point to the pivotal role that pragmatic RCTs play to overcome these challenges.

A central question is whether a meaningful distinction exists between research and clinical practice. Contemporary ethical frameworks distinguish research from practice in order to demarcate the activities that ought to undergo review for the protection of research participants [13]. The term “research” designates “an activity designed to test an hypothesis, permit conclusions to be drawn, and thereby develop or contribute to generalizable knowledge;” whereas the term “practice” refers to “interventions that are designed solely to enhance the well-being of an individual patient or client” [13].

Most authors are critical of the relevance of the research-practice distinction for pragmatic RCTs. For instance, Kass and colleagues list factors supporting the need for a research-practice distinction, including the idea that research participation involves more risk to patients than clinical practice [21]. They argue, however, that this distinction is irrelevant because pragmatic RCTs commonly compare treatments used routinely in clinical practice and pose few additional risks to participants. Indeed, Faden and colleagues propose a new ethics framework for pragmatic RCTs according to which low-risk studies are to be governed by rules similar to those in clinical practice, and this approach enjoys considerable support in the literature [15, 16, 21–24].

Only a minority of authors support the need for a research-practice distinction. For example, Largent and colleagues write, “the moral distinction between human experimentation and standard medical care is… still relevant despite empirical data suggesting that the risk-benefit ratios of these practices often converge” [47]. In support of this claim, they argue that the research-practice distinction helps to distinguish between risks that are morally permissible and those that are morally impermissible: in research, risks are justified by the potential benefits to future patients; whereas, in practice, risks must be offset by direct benefits to patients [47].

#### Consent

The ethical principle of respect for persons requires researchers to take seriously the decisions of autonomous people and protect those who lack autonomy. This principle grounds the widely held presumption that people have a right to be free of experimentation without their consent [48]. Accordingly, researchers have an obligation to seek the voluntary informed consent of study participants [25]. (The specific elements of informed consent are discussed in the next section.)

While informed consent serves to further participant autonomy, the majority of authors espouse a different understanding of the purpose of consent. On their view, consent is required because of the added risks that research poses compared to clinical practice. While this view may not be stated explicitly, it is reflected in statements such as “complex informed consent documents… should not be required, nor are they desirable in low-risk situations” [41]. When participation in pragmatic RCTs poses low risk, most authors endorse either simplified consent or no consent. Table 2 summarizes different consent models discussed in the literature.

**Table 2:** Models of informed consent

Consent model	Characteristics	Authors
Standard informed consent	• Written disclosure of all information • Consent obtained in writing	Anderson & Schonfeld 2014
Targeted consent model	• Verbally disclose information • Obtain written consent	Wendler 2015
Integrated consent model	• Verbally disclose information • No written consent required • Physician documents consent in electronic health record	Kim & Miller 2014
Streamlined consent	• No consent is sought	Faden, Kass, Whicher, Stewart, & Tunis 2013

Faden and colleagues argue for a “streamlined consent” process [16]. They acknowledge that under current regulations informed consent is required for pragmatic RCTs; however, they imagine a future set of structures within a learning health care system, including ethics oversight panels and public notification, that would allow for the elimination of consent in many cases. They ground their view in the Rawlsian notion of “common purpose,” according to which people will cede autonomy rights due to a shared public commitment to promote the ends of the health system. From this perspective, pragmatic RCTs that pose minimal risk and are approved by ethics oversight panels could proceed without consent [16, 24].

McKinney and colleagues argue for an expanded use of a waiver or an alteration of consent in pragmatic RCTs [26]. They believe that many pragmatic RCTs may proceed with a waiver, in which “no attempt is made to notify or request permission from research subjects prior to participation” [26]. A waiver may be granted by a research ethics committee when study participation poses only minimal risk and it would be infeasible to conduct the study with informed consent. Therefore, this approach is “limited to cases in which the risks of participation are low and where more engaged consent options are unworkable” [26].

Other authors argue that simplified (or “altered”) informed consent procedures are more appropriate for pragmatic RCTs. These approaches seek to balance the need to obtain informed consent with associated burdens, including cost, time, and complexity [27, 28, 41]. Kim and Miller propose an “integrated consent” model in which verbal consent for research is obtained in a clinical setting [27, 29]. According to their approach, the physician engages the patient in a conversation about trial participation. If the patient agrees to participate, the “physician does what she would ordinarily do in the course of her practice—that is, document the interaction” [26]. Wendler proposes a slightly different approach, called “targeted consent,” which contains both verbal and written components [28]. In targeted consent, the patient is required to sign a consent form after the conversation about pragmatic RCT participation.

A minority of authors advocate for preserving standard consent requirements. Anderson and Schonfeld argue that “patients have the right to choose whether to participate [in pragmatic RCTs] after they have been informed… irrespective of the risk levels of the intervention” and believe that “written documentation of the patient’s agreement to be a research subject remains essential” [30]. Elsayyad agrees, saying that “the doctrine of informed consent is intended to protect a patient’s decision-making ability and to respect the person’s dignity” [31]. Modi worries that forgoing the standard informed consent process would differentially impact patients who have poor health literacy [32]. Finally, Menikoff points out the proposals to eliminate informed consent entirely “are surprisingly sketchy on the details about which clinical trials would still require informed consent” [33].

#### Disclosure

Current regulations require that prospective research participants receive: a statement that the study involves research; an explanation of its purpose and procedures; a description of reasonably foreseeable risks or discomforts; a description of possible benefits to the participant or society; a description of alternative treatments; an explanation of steps taken to protect the confidentiality of private health information; for research posing more than minimal risk, an explanation as to whether compensation will be available for research related injury; an explanation of whom to contact about the research or the participant’s rights; and, finally, a statement that research participation is voluntary and study refusal (or withdrawal) will not result in the loss of benefits to which the participant has a right [49]. Among authors who believe that informed consent ought to be obtained, there is some disagreement as to what needs to be disclosed to participants in low-risk studies. Authors generally agree that the research purpose, risks and benefits and voluntary participation must be disclosed, but they disagree as to whether randomization must be disclosed [27, 28, 34–39].

##### Disclosing research purpose

Authors agree that the purpose of the research ought to be disclosed to prospective participants [28, 34–36, 39]. Spellecy and colleagues state that clinicians should disclose the purpose of a pragmatic RCT, as not doing so would mean that participants “have not been afforded the opportunity to agree to the ends of the research and, to some extent, make those ends their own” [35].

##### Disclosing risks and benefits

Among those who support the need for informed consent, there is also agreement that the risks and benefits of participation in a pragmatic RCT must be disclosed [27, 28, 34–39]. Wendler states it is necessary to “disclose any added risks, disclose any information that is important for most subjects, and then discuss with the individual any questions or concerns that they may have” [28]. In a previous paper, Wendler makes clear that by “added risks” he refers only to the incremental risks of research participation [36].

##### Disclosing voluntariness

The authors who discuss the voluntary nature of participation in a pragmatic RCT believe that it needs to be disclosed [27, 28, 34]. For instance, Wendler says that consent requires an explicit statement that study participation is voluntary—e.g., “whether you participate is up to you”—and that participants can decline or withdraw their participation [28]. Kim and Miller state that their integrated consent model “lacks explicit statements regarding voluntariness,” but it must be part of a conversation about the trial [27].

##### Disclosing randomization

Authors disagree as to whether randomization needs to be disclosed. The discussion of randomization focuses on its impact on the physician-patient relationship. Those who believe that randomization need not be disclosed in some cases focus on properties of the treatments to which patients are allocated; those supporting disclosure highlight how randomization removes the personalized judgment of the physician—an important protection for patients.

According to Wendler “when existing data do not suggest that one intervention is better, randomization does not increase risk” [28]. Accordingly, “for the purpose of protecting subjects, [pragmatic RCTs] do not need to disclose randomization.” Feudtner and colleagues agree that randomization does not add risk and disclosure is not required [38].

Other authors disagree and believe that randomization must be disclosed to prospective research participants [27, 34, 39]. For instance, Kim and Miller state that they “find the proposal not to disclose to patients the fact of randomization even in low-risk [pragmatic RCTs] problematic for both ethical and practical reasons” [27]. On their view, the act of concealing this information from the patient is impermissible. O’Neil’s stance is that randomization should be disclosed so that a patient does not form the mistaken belief that a given treatment was recommended by the physician [39].

#### Oversight

##### Oversight burdens

The reviewed literature describes research oversight as burdensome and a barrier to conducting pragmatic RCTs. Research ethics committees are conceived as causing delays and hindering the overall progress of research with little added value. Richard Platt and colleagues express this view when they say, “[t]he so-called protections being debated reflect a view that informed consent is required for most activities labeled clinical research…The irony deepens when consent requirements become barriers to even low-risk studies intended to identify strategies to protect patients” [22].

The review process for low-risk pragmatic RCTs is considered time consuming, costly, and overly complex. But why is this the case? Kass and colleagues believe that these burdens are the result of RECs relying on the “faulty research-practice distinction as the criterion that triggers ethical oversight” [21]. Other authors ascribe the burdens of oversight to a lack of standardization among review committees [12, 50]. Within a given institution, a pragmatic RCT may be subject to the potentially competing demands of committees overseeing research ethics, grants and contracts, conflicts of interest, patient safety and pharmacy. Multisite pragmatic RCTs typically require review by multiple local RECs, each with its own submission forms, procedures and local practices. Accommodating potentially conflicting demands from multiple committees is a major challenge for researchers. O’Rourke and colleagues point out that the “lack of inter-institutional standardization often results in different review outcomes for the same protocol that can delay study operations from start-up to study completion” [12].

##### Appropriate oversight

Burdens and delays in the current system and the need for reform of research oversight for pragmatic RCTs is a major theme. Most authors believe that the review process must be streamlined [40–44]. Some authors focus on steps to improve the current review process across the board, including transparent policies, safeguards and stakeholder participation [51, 52]. Some suggest the need for a case-by-case approach to determine when streamlined review is both needed and appropriate, depending on the level of risk posed [44, 53, 54].

A minority of authors advocate for a more radical solution. Faden and colleagues call for the abandonment of the current review process in exchange for a new oversight system for low-risk pragmatic RCTs [15]. They argue that the transition to the learning health system requires new oversight mechanisms and policies. In place of RECs, they propose “oversight committees” that will assess harms and benefits, consider the need for individual consent, and notify the public when consent is not obtained.

## Discussion

Our review of the ethics of pragmatic RCTs literature found that six of the seven articles that discuss the distinction between research and practice reject it. These authors argue that the reasons for distinguishing research from practice are inadequate and based upon faulty assumptions. Their arguments seemingly rest upon the assumption that the research-practice distinction exists to protect research participants from risk. They point out that clinical practice may sometimes pose greater risk than research. Moreover, they argue that the research-practice distinction is particularly outmoded for pragmatic RCTs, as they contain elements of both research and practice. Finally, they argue that the research regulations to which the distinction has given rise are overly cumbersome for low-risk pragmatic RCTs and, as a result, stifle socially-valuable research.

It is perhaps remarkable, then, that authors have failed to correctly identify the reason for treating research and practice as morally separate domains. The distinction is not driven by the need to protect research participants from higher levels of risk. Rather, the research-practice distinction stems from the differences in the relationships between physician and patient, on the one hand, and researcher and participant, on the other. Clinical practice is characterized by the trust relationship between physician and patient. The physician owes the patient duties of loyalty, discretion and personal care [55]. However, research introduces competing interests—e.g., the production of generalizable knowledge, financial compensation, career advancement, and reputation—and these competing interests undermine the physician’s unfettered loyalty to the patient. Combining clinical practice activities with research may also lead to cases of therapeutic misconception; that is, patients may mistakenly believe that research activities are meant to directly benefit them. Acknowledging the conflicts of interest inherent in clinical research, research regulations and review by RECs serve as “strangers at the bedside”—independent mechanisms to protect the interests of patients in research [56].

The question as to whether pragmatic RCTs pose additional risks to participants is therefore orthogonal to whether the research-practice distinction is meaningful. Even if research poses the same—or even a lesser— degree of risk compared to clinical practice, the protective mechanisms of regulation and independent review are required. From the standpoint of ethical principles, all research involving human participants falls within the purview of research regulations and RECs. “Research” is traditionally defined as a class of activities designed to contribute to generalizable knowledge [25]. “Human participants” are those who are the direct target of study interventions [25, 49]. (It should be noted that some national research regulations may exempt from review certain activities that fulfill these definitions). Rather, the central question at issue should be: how ought low-risk pragmatic RCTs be regulated and reviewed?

Given that pragmatic RCTs meet the definition of research involving human participants, we believe that the answer to this question must appeal to internationally accepted research ethics principles. Shifting discussion to ethical principles can offer some broad guidance to pragmatic RCTs (see Table 3). First, the principle of respect for persons requires, on one interpretation, researchers to treat prospective research participants as ends and not merely as means to an end. All research involves exposing participants to risk for the benefit of others. It is only through the informed consent process that the participant adopts the ends of the study as her own, and thus is treated as an active partner in research. This explanation helps make clear the centrality of consent to the ethics of research. Proposals to eliminate consent merely when participation in pragmatic RCTs poses low risk fail to show due regard for the liberty interests of participants. Waivers of consent require justification: the research must be socially important; requiring informed consent would make the study infeasible; and, participation poses only minimal risk [25]. We believe that further work is required to operationalize the notion of study “infeasibility” in the context of pragmatic RCTs. To what degree do cost, time, and the complexity of information constitute a justification for a waiver?

**Table 3:** Critical analysis of the major themes and gaps identified

Main themes	Critical Analysis of the Dominant View	Gaps in the Literature
Research-practice distinction	• The rejection of the research-practice distinction rests on a mistaken assumption that the classification turns solely on risk. • Pragmatic RCTs are research, so a better starting place is oversight for other categories of low-risk and socially important research.	• Given that pragmatic RCTs are research, what are the implications for consent, disclosure, and oversight?
Consent	• The central focus on risk leads to the spurious conclusion that consent need not be sought for all low-risk research. • The ethical principle of respect for persons requires that consent be sought unless conditions for a waiver apply.	• When are waivers appropriate? • What is meant by ‘impracticable’? • Does cost, time and complexity count as sufficient justification?
Disclosure	• Grounding the disclosure of information in risk is irrelevant to why information is disclosed. • The ethical principle of respect for persons requires that information be disclosed for research participants to compare their values with the details of the research.	• What type of notification is best for disclosing information? • Which approaches to notification uphold respect for persons to the highest degree?
Oversight	• The level of scrutiny from RECs need not be proportionate to the level of risk of the research. • Proportionate review ought to be based on several factors, including presence of vulnerable participants, degree of risk and methods. • Multisite trials ought to be reviewed by a central REC.	• What models for centralized review of multisite pragmatic RCTs are successful, and why?

Second, the ethical principle of respect for persons also guides the disclosure of information to prospective research participants [13]. The presumption in the literature that the disclosure of information ought to be based on incremental risk misses the point. Information about the research is disclosed to allow participants to evaluate the details of the pragmatic RCT against their own beliefs and values. A prospective research participant might ask a number of questions in this process: “Are the goals of the study important to me?”; “Are the risks acceptable in light of my own past experiences and preferences?”; and, “Are the study procedures, both in terms of treatments and data collection procedures, and their scheduling compatible with the other goals I seek to achieve?” Further work is required to guide the practice of notification in pragmatic RCTs. When and how should patients be notified of an ongoing pragmatic RCT and its results? Empirical research into the impact of differing approaches, such as posters, other advertisements and letters could usefully guide practice.

Third, we suggest proportionate review for all pragmatic RCTs. Proportionate review is based on the common-sense idea that the instensity of review ought to depend features of the study [57]. Pragmatic RCTs that involve vulnerable participants, high risk to participants (e.g., privacy and confidentiality risks, psychological stress, or burdens due to extra visits), or difficult methodological issues (e.g., flawed or highly complex designs) require more careful review. Those that do not involve vulnerable participants, pose low risk to participants and are methodologically sound may be reviewed in an expedited manner. Successful models for centralized review of multisite pragmatic RCTs should be examined and, where possible, implemented.

Fourth, the presumption in the literature that pragmatic RCTs are morally unproblematic because they are socially important, or may employ only usual care interventions and routinely collected data requires further examination. These trials are socially important only if the research question has real world relevance, and the trial is designed to generate high quality evidence with a high probability of direct uptake in practice [58]. Usual care interventions may pose minimal risk; however, there are circumstances in which pragmatic RCTs employ health policies or health services interventions with little or no evidence of efficacy [59], or with no direct benefit to patients, e.g., post-marketing trials that serve the interests of pharmaceutical companies rather than patients [60]. There is also no discussion in the literature on the risks of poor scientific value, or of the risks posed by conflicts of interest in the case of commercially sponsored pragmatic RCTs. And, as witnessed by recent development in the European Union, there are important consideration about patient data ownership within the electronic health record [61]. These are all important issues that require further discussion.

## Conclusion

While the social imperative for pragmatic RCTs to inform patients, providers and health system managers is global, the current discussion about the ethical issues in such trials overwhelmingly involves American voices. Indeed, 35 of the 36 articles included in our review were published in the United States by (predominantly) American authors. Overwhelmingly, the framework appealed to in these articles is that of U.S. federal research regulations. While the United States’ regulations are important to an American audience, they undercut the relevance of scholarship to a global audience and international ethical guidelines [13, 25, 57, 62, 63]. Shifting the conversation from national regulations to internationally accepted ethical principles would be salutary in several ways, including scaffolding a global conversation about an issue of international relevance. Developing international ethical standards for the design and conduct of pragmatic RCTs requires the participation of all.

## Abbreviations

RCT: Randomized controlled trial
REC: Research ethics committee
